# In Vitro Antiproliferative Activity of Echinulin Derivatives from Endolichenic Fungus *Aspergillus* sp. against Colorectal Cancer

**DOI:** 10.3390/molecules29174117

**Published:** 2024-08-30

**Authors:** Hind Makhloufi, Aline Pinon, Yves Champavier, Jennifer Saliba, Marion Millot, Ingrid Fruitier-Arnaudin, Bertrand Liagre, Guillaume Chemin, Lengo Mambu

**Affiliations:** 1 LABCiS, UR 22722, Faculté de Pharmacie, Univ. Limoges, F-87000 Limoges, France; hind.makhloufi@unilim.fr (H.M.); aline.pinon@unilim.fr (A.P.); marion.millot@unilim.fr (M.M.); bertrand.liagre@unilim.fr (B.L.); guillaume.chemin@unilim.fr (G.C.); 2 Univ. Limoges, CNRS, Inserm, CHU Limoges, BISCEm, UAR 2015, US 42, F-87025 Limoges, France; yves.champavier@unilim.fr; 3 Laboratoire LIENSs, Université de La Rochelle, UMR CNRS 7266, F-17000 La Rochelle, France; jennifer.saliba@univ-lr.fr (J.S.); ingrid.fruitier@univ-lr.fr (I.F.-A.)

**Keywords:** endolichenic fungi, *Aspergillus* sp., human colorectal cancer, chemoresistant cancers

## Abstract

The endolichenic fungus *Aspergillus* sp. was isolated from the lichen *Xanthoparmelia conspersa* harvested in France. *Aspergillus* sp. was grown on a solid culture medium to ensure the large-scale production of the fungus with a sufficient mass of secondary metabolites. The molecular network analysis of extracts and subfractions enabled the annotation of 22 molecules, guiding the purification process. The EtOAc extract displayed an antiproliferative activity of 3.2 ± 0.4 µg/mL at 48 h against human colorectal cancer cells (HT-29) and no toxicity at 30 µg/mL against human triple-negative breast cancer (TNBC) cells (MDA-MB-231) and human embryonic kidney (HEK293) non-cancerous cells. Among the five prenylated compounds isolated, of which four are echinulin derivatives, compounds **1** and **2** showed the most important activity, with IC_50_ values of 1.73 µM and 8.8 µM, respectively, against HT-29 cells.

## 1. Introduction

According to World Health Organization (WHO) reports, cancer constitutes a major health problem [1]. In the human body, healthy cells adhere to a highly regulated pattern of proliferation. Cell division is subject to stringent regulation and continuous monitoring to prevent abnormal proliferation; this constant coordination is essential for the proper functioning of the body. However, cancer cells deviate from these regulations, bypassing the normal controls over cell proliferation due to the deregulation of their division programs. This unchecked proliferation is often associated with the ability of certain cancer cells to migrate from their site of origin, detach, and travel through the bloodstream and lymphatic system. This leads to the invasion of surrounding tissues or even distant parts of the body, forming secondary tumors, a process known as metastasis [2]. Tumors formed by these malignant cells tend to become increasingly aggressive over time and can be life-threatening by disrupting the functions of the newly affected tissues and organs.

In 2020, 1.9 million colorectal cancer (CRC) cases were diagnosed, leading to around 900,000 deaths [3] CRC can develop in the four main parts of the colon: the ascending colon, the descending colon, the transverse colon, and the rectum. It often starts as benign polyps that may become cancerous over time. Further, breast cancer was the most common cancer in 157 out of 185 countries, with 2.3 million cases and a 6.9% mortality rate [3]. Triple-negative breast cancer (TNBC), a particularly aggressive and chemoresistant subtype, lacks three key receptors, i.e., estrogen receptor alpha (Erα), progesterone receptors (PRs), and HER2, making endocrine therapy ineffective [4]. This subtype has a high recurrence and low survival rate. Treatment options include surgery, radiotherapy, chemotherapy, and immunotherapy, which are effective in early stages but less so later, often causing severe side effects, recurrence, and resistance in the case of CRC [5].

The progress in in biology, immunotherapy and the substantial improvements in modern drug design and manufacturing have made the discovery of a cure for cancer a feasible goal [6]. Despite the significant advances of current therapies, multiple side effects and adverse reactions in normal cells have been reported with chemotherapy, including nausea, vomiting, mucositis, alopecia, neuropathy, and myelosuppression [7]. Furthermore, they have been found to be associated with multidrug resistance (MDR), an undesirable phenomenon responsible for more than 90% of the deaths of cancer patients undergoing chemotherapy [7].

These observations motivate the search for other effective cures with fewer side effects constituting better alternatives that balance efficacy and toxicity and comply with drug resistance prevention measures.

Natural products represent an available source of new drugs, drug leads, and chemical entities [8]. Approximately 80% of the approved chemotherapeutic drugs [9] and more than half of all drugs are based on bioactive natural products [10]. Eighty-seven percent of human diseases, including cancer, are treated by using natural products [11]. Natural bioactive molecules exhibit cytotoxic effects by attacking macromolecules expressed by cancer cells, such as those in oncogenic signal transduction pathways [12]. They exhibit several advantages: they are mainly non-toxic or present little toxicity, are highly compatible, are associated with multitargeted actions, and can constitute the base for lead compounds [8]. Finally, some have demonstrated abilities to enhance common anticancer therapies’ efficacity by lowering the effective doses of cytotoxic drugs or overcoming their limitations, like in colorectal cancer immunotherapy [13].

Thus, it is important to search for other therapeutic agents with more innovative means, notably through natural bioactive substances [14]. The genus *Aspergillus* comprises several hundreds of species of mold that are remarkably diverse and adaptable to a wide range of the planet’s environments. These species are important from an ecological and economic standpoint in many ways. Certain *Aspergillus* species are beneficial, especially for industrial uses, but others are very dangerous to human health due to the production of mycotoxins [15].

Like plants, lichens provide an enabling environment for various microscopic organisms [16]. Among them, endolichenic fungi (ELF), which are different from lichen mycobionts and lichenicolous fungi, live asymptomatically in the lichen thallus. They resemble endophytes in plants [17].

Endolichenic fungi constitute a niche for secondary metabolites, and an original source for the discovery of new natural substances of therapeutic interest [18]. Over the last few decades, researchers have become increasingly interested in endolichenic fungi.

Chemical investigations on ELF highlighted their ability to biosynthesize interesting metabolites, of which 64% were isolated as new compounds [14]. High chemical diversity is described in ELF, as they produce alkaloids, peptides, polyketides, polyphenols, terpenoids, steroids, and others, like fatty acids and chain alcohols [14,18]. These molecules have been demonstrated to have therapeutic potential as antibacterial immunosuppressive, antiviral, antiparasitic, anticancer, antifungal, and anti-Alzheimer’s agents [19,20,21,22].

As part of our ongoing search for potential anticancer compounds from endolichenic fungi, we have evaluated the antiproliferative activity of a pool of extracts obtained from different endolichenic species. Preliminary screening in our laboratory showed that the EtOAc extract of *Aspergillus* sp. (XC04 GP) isolated from the lichen *Xanthoparmelia conspersa* exerted substantial in vitro activity against HT-29 human CRC cell line, whereas it showed no cytotoxicity against human TNBC nor non-cancerous HEK293 (human embryonic kidney) cells.

This article deals with the isolation and structural elucidation of bioactive compounds from *Aspergillus* sp. The antiproliferative activity of the isolated compounds was evaluated against the HT-29 cell line after MS- and bio-guided fractionation from crude EtOAc extract.

## 2. Results

### 2.1. Fungal Identification, Culture, and Extraction

The endolichenic fungus *Aspergillu*s sp. was obtained from the culture of the lichen *Xanthoparmelia conspersa* in Sabouraud medium. Molecular identification was performed by comparison of its DNA sequence with those present in the GenBank database. The similarity of its ITS sequence enabled only the determination of the genus.

The culture of *Aspergillus* sp. was carried out in a solid culture medium on a small scale (400 mL) and a large scale (6 L) by using potato dextrose agar (PDA). After 21 days at controlled temperature (25 °C), extraction was performed, and the resulting EtOAc crude extracts XC04 P and XC04 GP were obtained from the small- and large-scale cultures, respectively.

Liquid–liquid partition was performed from the EtOAc crude extract (XC04 GP) with hexane and aqueous methanol (10:90) in order to remove the fatty acids abundantly biosynthesized by the fungus. This resulted in hexane and hydro-methanol subfractions along with an intermediate extract and a precipitate.

### 2.2. Viability Test

As multidrug resistance (MDR) is responsible for over 90% of deaths in cancer patients receiving traditional chemotherapeutics, two chemoresistant cancer cell lines were selected, i.e., HT-29, a cell line derived from colorectal adenocarcinoma and MDA-MB-231, a cell line derived from triple-negative breast cancer, for the evaluation of antiproliferative activity. In parallel, the non-cancerous HEK293 cell line, isolated from the kidney of a human embryo [23], was tested as a control.

The EtOAc extracts resulting from the small-scale and the large-scale cultures on PDA showed similar activity at 24 and 48 h against the HT-29, MDA-MB-231, and HEK293 cell lines.

**Figure 1:**
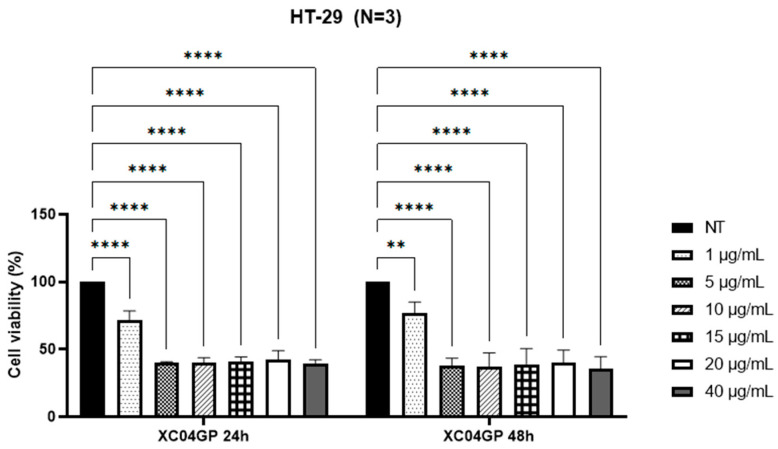
Percentage of viability of HT-29 cells after treatments with XC04 GP extract at 24 h and 48 h. ** *p* < 0.01; **** *p* < 0.0001; NT: Not Treated.

**Figure 2:**
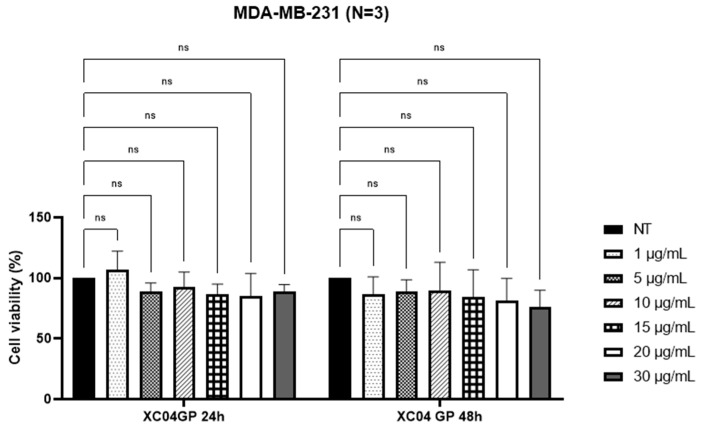
Percentage of viability of MDA-MB-231 cells after treatments with XC04 GP extract at 24 h and 48 h. NT: Not Treated; ns: not significant.

**Figure 3:**
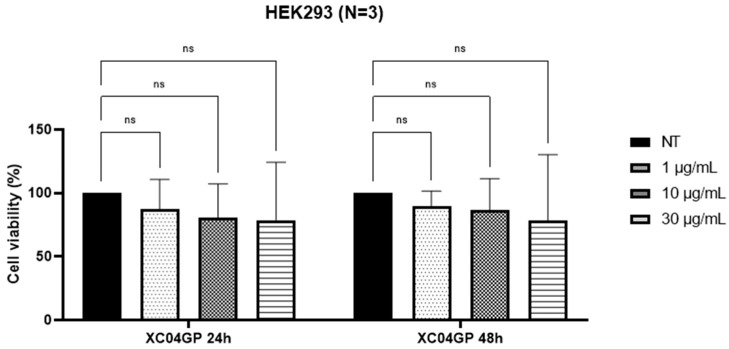
Percentage of viability of HEK293 cells after treatments with XC04 GP extract at 24 h and 48 h. NT: Not Treated; ns: not significant.

As shown by the MTT results (Figure 1), XC04 GP displayed selectivity towards HT-29 cells, with IC_50_ of 2.90 ± 0.4 µg/mL at 24 h and 3.20 ± 0.4 µg/mL at 48 h. However, it was not active at the maximum concentrations tested (30 µg/mL) against MDA-MB-231 cells (Figure 2). This extract had no toxicity against the HEK293 cell line at 30 µg/mL (Figure 3).

By following a bio-guided approach, the growth inhibition of HT-29 cells after treatment with the resulting subfractions and precipitate from the liquid–liquid partition of the XC04 GP extract was determined. The results of the MTT assay are summarized in Table 1.

The MTT test showed that the activity was preserved in both the precipitate and the intermediate subfraction, while the hexane and hydro-methanol or aqueous MeOH subfractions had moderate activity compared with XC04 GP. This confirms the importance of this step to eliminate the fatty acids synthetized by the fungus, which can mask the molecules’ activity.

### 2.3. Molecular Network

**Figure 4:**
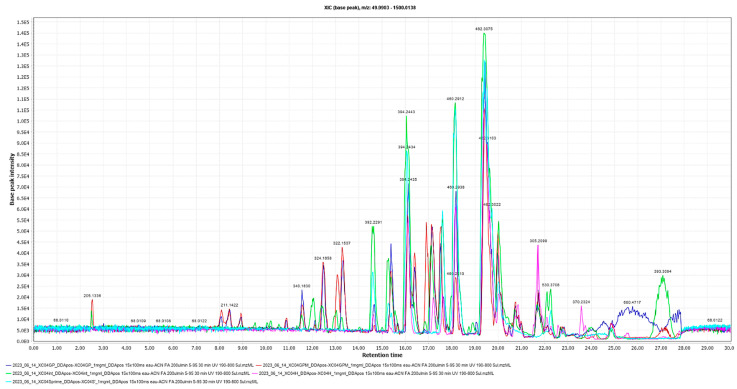
Comparison of LC/MS-MS chromatographic profiles in positive mode of extracts XC04 GP (large scale), XC04 GP-M (hydro-methanol subfraction), XC04 GP-H (hexane subfraction), XC04 GP-I (intermediate subfraction), and XC04 GP-S (precipitate).

The extract (XC04 GP) and the three other subfractions obtained from the partition were analyzed by LC/MS-MS in (Figure 4).

In this study, the MS-guided approach was also used to analyze and annotate the molecules composing the extract and their subfractions based on the comparison of their MS^2^ spectra with those listed in natural substance databases. The small-scale and large-scale extracts, as well as the hexane, hydro-methanol, and intermediate subfractions, and the precipitate were analyzed by using LC/MS-MS in positive and negative modes to generate a molecular network. The recovered data were converted into mzML format by using MSConvertGUI (64 bits) software (version: 3.0.19124-6efa8cbfe). The files were then processed by using MZmine 2.53 software, and depending on the parameters set, a quant file was generated. This file was then downloaded into MetGem software (version: 1.2.1), which was used to generate a molecular network. Molecule annotation was performed after downloading the databases, and the results given are based on a comparison of MS/MS fragmentation similarity between spectra among chromatograms. The similarity score (cosine) can be set from 0 to 1 (the closer to 1, the more reliable the similarity).

**Figure 5:**
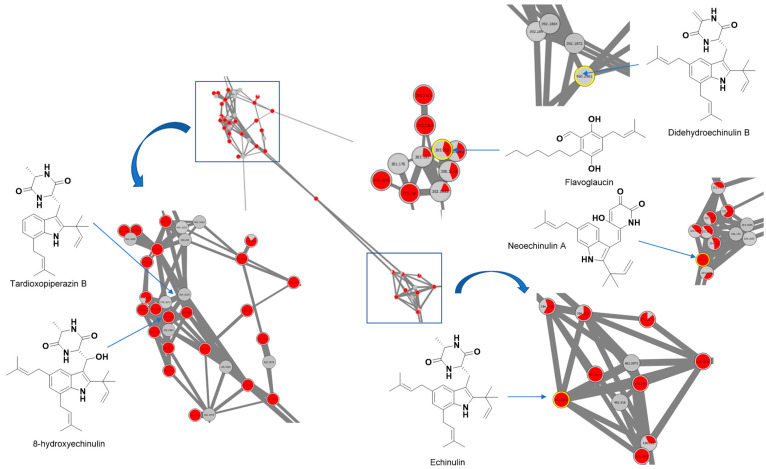
The molecular networks in positive mode of *Aspergillus* sp. extracts obtained by using MetGem software and some annotated compounds. Active extract and subfractions (red: XC04 GP, XC04 GP-I, and XC04 GP-S) and inactive extract and subfractions (gray: XC04GP-H and XC04 GP-M).

Few molecules were annotated from the negative-mode analysis. Thus, the annotations of data obtained in positive mode were favored. In this analysis, 67 hits corresponding to 22 molecules were annotated with a cosine score ≥ 0.7 and a minimum of four common fragments (Table 2). The annotated molecules can be classified into several categories, including cyclic peptides, phthalates, acylglycerols, amino acid derivatives, fatty acids and amides, and diketopiperazines. Among the molecules detected, echinulin at *m*/*z* 462.3056 [M + H]^+^ with a cosine of 0.7 was found in the active fractions, and flavoglaucin at *m*/*z* 305.2082 [M + H]^+^ with a cosine of 0.84 was found in both the active extract and corresponding active subfractions, giving indications for the purification process that led to the isolation of these two molecules (Figure 5). Several peaks were not annotated, such *m*/*z* 394.2515 and *m*/*z* 478.3012. Moreover, neoechinulin A was annotated at *m*/*z* 324.1679. This prompted us to purify and isolate the corresponding compounds.

However, the effectiveness of this technique depends to a large extent on the ionization and fragmentation of the compounds, as well as on the specific parameters used during analysis. Optimizing these parameters is crucial to obtaining reliable and accurate results. Furthermore, the databases available in the GNPS (Global Natural Products Social Molecular Networking), which facilitate direct identification by matching MS/MS spectra, are currently still limited in terms of the diversity and number of annotated molecules. This limitation hinders the ability to exhaustively identify compounds present in the analyzed extracts and subfractions. Nevertheless, this dereplication method offers valuable complementary information that enriches conventional phytochemical analysis. However, due to the limited number of molecules annotated from the molecular network, this approach was combined with the bio-guided fractionation of extracts and the purification of active fractions for the isolation of compounds.

### 2.4. Fractionation and Purification of Active Compounds

**Figure 6:**
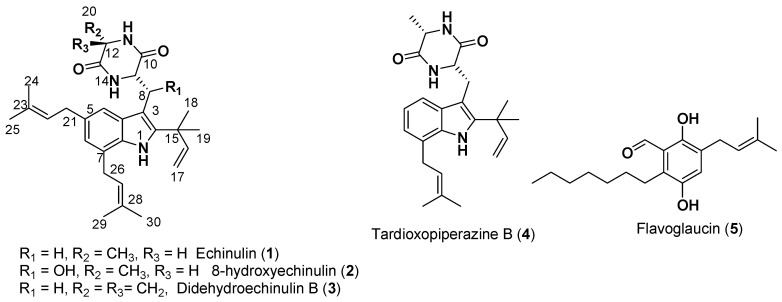
Structure of the isolated molecules from *Aspergillus* sp.

The XC04 GP extract underwent partitioning, and both the intermediate subfraction and precipitate were submitted to purification. Five prenylated compounds were isolated, including one hydroquinone and four diketopiperazines (Figure 6). Their structures were elucidated by careful analysis of their NMR spectra 1D (^1^H and ^13^C) and 2D (COSY, HSQC, and HMBC), HR ESI MS-MS data, and comparison with the spectroscopic data available in the literature. Among them, four compounds were already known and compound **2** was recently described [24]. Compounds **1**–**5** were identified as echinulin (**1**) [25], 8-hydroxyechinulin (**2**) [24], didehydroechinulin B (**3**) [26], tardioxopiperazine B (**4**) [27], and flavoglaucin (**5**) [28], respectively.

Compounds **1**, **2**, and **4** were isolated from the precipitate. Compounds **3** and **5** were isolated from the intermediate subfraction along with compounds **1**, **2**, and **4**. We noticed that echinulin (**1**) was the major compound found in the active intermediate subfraction and precipitate.

### 2.5. Antiproliferative Activity

The isolated compounds (**1**–**5**) were evaluated for their antiproliferative activity against HT-29 cells. Those molecules were not tested on MDA-MB-231. The results of the MTT assay are summarized in Table 3.

Echinulin (**1**) clearly displayed the highest activity against HT-29 cells, even more than 5-FU and irinotecan, with IC_50_ of 1.51 and 1.73 µM at 24 and 48 h, respectively. 8-Hydroxyechinulin (**2**) and tardioxopiperazine B (**4**) displayed interesting activity with IC_50_ values of 8.80 µM and 13.70 µM at 48 h, respectively. From all the echinulin derivatives, didehydroechinulin B (**3**) was the least active, with an IC_50_ of 44.84 µM at 48 h. Flavoglaucin (**5**) activity at 24 h was more significant than the positive control, with an IC_50_ value of 9.17 µM; however, at 48 h, the activity of flavoglaucin appeared to be closer to irinotecan, with an IC_50_ of 34.40 µM.

### 2.6. Study of Apoptosis

#### DNA Fragmentation

Apoptosis is a genetically programmed process of cell death which allows the cell to be eliminated after having received intra- or extracellular signals in order to maintain tissue homeostasis [29]. The cell can, therefore, induce the degradation of the genetic material (DNA fragmentation), which is one of the major markers of the phenomenon of apoptosis. In this study, the photometric ELISA for cell death detection was used for the in vitro quantitative determination of cytoplasmic histone-associated DNA fragments (mono- and oligonucleosomes) following induced cell death. The amount of DNA fragmentation under control conditions was normalized to 1, and that under treatment conditions was expressed as n-fold compared with the control.

**Figure 7:**
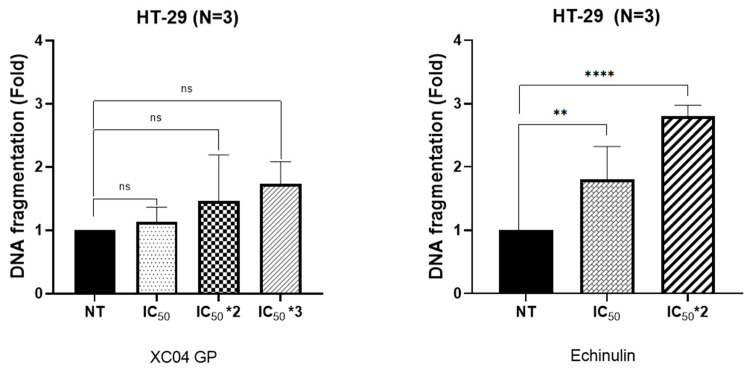
Quantification of DNA fragmentation after treatment with different concentrations of EtOAc extract (XC04 GP) and echinulin (ELISA test) on HT-29 cells. ** *p* < 0.01; **** *p* < 0.0001: ns: not significant.

Below, we show the DNA fragmentation fold results after treatment with the XC04 GP extract and echinulin (as a major isolated molecule) at IC_50_, IC_50_ × 2, and IC_50_ × 3 in HT-29 cells (Figure 7).

For both the EtOAc extract and echinulin (**1**), DNA fragmentation was often visible in a concentration-dependent manner. The MTT assay results are confirmed by DNA fragmentation, whose results were 1.7- and 2.7-fold for XC04 GP and echinulin treatments, respectively, at the IC_50_ × 3 and IC_50_ × 2 concentrations.

## 3. Discussion

Natural products are recognized as a niche of bioactive compounds that could lead to the discovery of new anticancer drugs and that have led to the successful development of numerous naturally derived anticancer agents into drugs over the last 30 years [30]. Secondary metabolites from lichens and endolichenic fungi have also been demonstrated to have a wide range of anticancer activities against various types of cancers [31,32,33,34,35,36,37].

Some endolichenic fungal species may produce selected types of compounds. Environmental factors including pollution, biodiversity loss, and climate change might also have an impact on this production. For instance, the genus *Aspergillus* is known to biosynthesize some unique secondary metabolites (asperglaucide, asperentins, auroglaucins, echinulins, epiheveadride, flavoglaucins, and neoechinulins) [38].

Apoptosis is the process of programmed cell death and the most popular target of many anticancer therapies. The dysregulation of apoptosis signals in cancers promotes abnormal cell growth and tumorigenesis [39]. Restoring the lost apoptotic function in cancer cells is the main objective of much of the research on anticancer drugs. The initiation of apoptosis in cells can be identified by morphological changes, such as nuclear fragmentation, chromatin condensation, cell shrinkage, and membrane blebbing [40].

The EtOAc extract (XC04 GP) showed an antiproliferative effect on CRC cells (HT-29) with an IC_50_ value of 3.2 ± 0.4 µg/mL and insensitivity towards TNBC cells (MDA-MB-231) when treating at 30 µg/mL, and the proliferation of HEK293 cells was not affected by treatment at this same dose. This result suggests that the extract XC04 GP is not toxic to that non-cancerous cell line, which is a promising result and highlights a potential selectivity toward this type of chemoresistant colorectal cells. Thus, the chemical investigation of the extract is relevant in order to isolate molecules responsible for this activity, which are more likely to be less toxic than actual anticancer drugs. These results are confirmed by the DNA fragmentation of HT-29 cells after treatment with different concentrations of the *Aspergillus* sp. EtOAc extracts, showing a slight increase in DNA fragmentation, 1.7-fold compared with the control cells.

The molecular network resulted from the LC/MS-MS analysis of the EtOAc extracts and their subfractions (hydro-methanol, hexane, and precipitate) enabled the annotation of some of the most common compounds for this genus, the echinulin derivatives and flavoglaucin [41]. Moreover, amino acid derivatives and cyclic peptides were also highlighted with other compounds. The annotation was limited but focused on compounds responsible for activity, such as echinulin, neoechinulin A, and flavoglaucin, which were annotated in the EtOAc extracts, intermediate subfraction, and precipitate.

Several other molecules present in the active extracts and subfractions were not annotated, such as isolated compounds **2**, **3**, and **4** at *m*/*z* [M + H]^+^ 460.2964, *m*/*z* [M + H]^+^ 478.3069, and *m*/*z* [M + H]^+^ 394.2515, respectively. Their detection seems difficult because their MS^2^ spectra have not yet been included in the databases. However, this work also shows that this ELF biosynthesizes several compounds in very low quantities, which makes their purification difficult despite their annotation by LC/MS-MS.

In 1948, echinulin (**1**) was isolated for the first time from the mycelium of *Aspergillus echinulatus* [42]. Afterwards, echinulin was isolated from *Eurotium repens* cultured on MEA or PDA medium [15,43] and also from *Aspergillus chevalieri* grown on yeast extract sucrose medium [44]. In this study, echinulin was isolated from an endolichenic fungus, *Aspergillus* sp., grown on PDA medium and was isolated as a major compound (26%) from the active extracts. The antiproliferative activity of echinulin against HT-29 cells was evaluated, and it was clearly significant compared with two anticancer drugs commercialized for use in colorectal cancer treatments. 5-Fluorouracil (5-FU) is a first-line treatment for many cancers, including CRC. 5-FU leads cells to death by preventing DNA replication and RNA synthesis through the inhibition of cellular thymidylate synthase [45,46], while irinotecan, known by the commercial name Campto^®^, targets topoisomerase 1 to treat metastatic or advanced tumors such as colorectal cancer [47]. Echinulin clearly had the highest activity on HT-29 at 48 h, almost 10 times more than 5-FU and 16 times more than irinotecan. In addition, Yan et al. demonstrated that the immunizing effect of echinulin on T cells is enhanced by the activation of the NFĸB signaling pathway, suggesting that it could serve as a novel immunotherapeutic agent [48]. Smetanina et al. have also demonstrated the cytotoxicity of echinulin on three different types of human prostate carcinoma (22Rv1, PC-3, and LNCaP) cell lines with IC_50_ values of 63.20, 41.70, and 25.90 µM [49]. It exhibited low cytotoxicity towards the Hela cell line [42]. Echinulin is also considered an antioxidant agent, with an IC_50_ of 18 µM, more active than ascorbic acid, with an IC_50_ of 25 µM [50]. In this study, it was proven that echinulin induces apoptotic process in HT-29 cells by causing DNA fragmentation with a fold of 2.7 after treatment with IC_50_ × 3, and this result comes to complete its antiproliferative activity against chemoresistant colorectal cancer.

8-Hydroxyechinulin (**2**) was isolated for the first time just recently from *Aspergillus amstelodami* BSX001 grown on modified PDA medium for 5 days and then on Czapek’s medium for another 4 days [24]. In this previous work, the antioxidant activity of this compound was described. In this study, we isolated 8-hydroxyechinulin from the endolichenic fungus *Aspergillus* sp. grown also on PDA medium. To the best of our knowledge, this study reports, for the first time, the activity of 8-hydroxyechinulin against HT-29 cells. Its activity on the growth inhibition of chemoresistant cells as HT-29 was interesting, with an IC_50_ of around 8.8 µM. This activity is twice higher than 5-FU and three times better than irinotecan, which is promising, and further biological test are needed to comprehend the signaling pathway and the toxicity of this molecule. The structure of compound **2** was found to have a hydroxyl group at C8, instead of a hydrogen as in compound **1**, which led to a slight reduction in activity.

Didehydroechinulin B (**3**) was first isolated from ocean fungus *Penicillium griseofulvum* grown on PDA medium [51] and was also isolated from the marine fungus *Aspergillus chevalieri* grown also on PDA medium [26]. Its production was also reported from the soil-derived fungus *Aspergillus effuses* H1-1 grown on PDA medium [52]. The antiproliferative activity against HT-29 cells was only determined at 48 h as being around 44 µM. In the literature, it was demonstrated that the activity of didehydroechinulin B was quantified as 4.20 µM and 1.43 µM in suppressing the proliferation of human hepatocarcinoma and lung cancer cells, respectively [53].

The structure of didehydroechinulin B (**3**) turned out to have a double bond at position C12, instead of a methyl group in the echinulin structure. This work highlights the impact of the presence of this methyl moiety at C12 for the activity, which was drastically reduced in comparison with echinulin (**1**).

Tardioxopiperazine B (**4**) was first isolated in 1999 from an Ascomycete, *Microascus tardifaciens*, grown on sterilized rice [27]. It was also isolated from the fungus *Aspergillus amstelodami* grown on malt plates [54]. In this study, the antiproliferative activity of tardioxopiperazine B was shown to be less than that of echinulin, with an IC_50_ of 13.7 µM at 48 h, against HT-29 cells, but still more interesting than 5-FU and irinotecan activities. A weak immunosuppressive activity was described for tardioxopiperazine B against the mitogen-induced proliferation of mouse splenic lymphocytes [27]. Also, tardioxopiperazine B displayed moderate antimelanogenic activity compared with echinulin, with IC_50_ values of 30 µM and 98 µM, respectively, against B16 melanoma cells [54]. Orfali et al. showed that echinulin and tardioxopiperazine B can act selectively against B16 melanoma cancer cells by inhibiting melanin synthesis, with IC_50_ values of 38.50 and 52.60 µM, respectively [54].

Based on a structure–activity relationship study and as previously reported [55], the presence of the prenyl groups at C-5 and C-7, the vinyl moieties at C-15 to C-17, and the carbonyl groups at C-10 and C-13 are of great importance for activity. In this study, this was confirmed, and echinulin activity against HT-29 was very interesting, with an IC_50_ value of 1.73 µM. On the other hand, the activity of tardioxopiperazine B dropped and was 7.91 times lower when it only had one prenyl group at C-7 compared with echinulin. Thus, this study highlights that the presence of the two prenyl groups is essential for antiproliferative activity.

According to these findings, the antiproliferative activity of the four echinulin derivatives can be affected by the presence or the substitution of some groups. The substitution of a proton at position C8 can slightly decrease the antiproliferative activity, as shown for compound **2**. The presence of two prenyl groups at positions C5 and C7 enhances this activity, and the presence of a methyl group at position C12 turns out to be the key to echinulin’s highly intriguing activity against HT-29 cells.

Although pulmonary and hepatic toxicity has been demonstrated for echinulin in rabbits [44], this result paves the way for work on modulating its activity by the hemisynthesis of derivatives. Echinulin shows considerable promise in targeting specific, chemoresistant cancer cells, making it a valuable candidate for further investigation. Access to a large number of echinulin derivatives will enable to refine its structure–activity relationship, enhance its antiproliferative activity, and reduce its toxicity, with a view to possibly use it in therapeutics.

Flavoglaucin (**5**) was first isolated in 1949 from *Aspergillus glaucus* [42] and then from *Eurotium repens* S-669 grown on MEA medium [15]. Flavoglaucin is also known to be produced by several species of the genus *Aspergillus*, like *Aspergillus amstelodami* and *Aspergillus calidoustus*, grown on PDA medium [44,56,57]. In this study, flavoglaucin was isolated from the endolichenic fungus *Aspergillus* sp. The antiproliferative activity against HT-29 cells was 34.40 µM, which is interesting. Flavoglaucin has a great impact in vitro on human epithelial colorectal adenocarcinoma (Caco-2), lung cancer (A549), and human breast carcinoma (MCF-7) cell lines, with IC_50_ values of 2.87 and 22.20 µM and a percentage of inhibition of 27 ± 0.53% (at 10 µM), respectively [28,57]. In vivo, Yoshimi et al. demonstrated that the multiplicity and the incidence of intestinal tumors are lower in rats given flavoglaucin with azoxymethane; thus, flavoglaucin, as a natural product, can be a promising chemopreventive agent for human large-bowel neoplasia [58]. The comparative cytotoxic activity of prenylated benzaldehyde derivatives revealed that both the position of the double bond in the prenyl group at C-3 and the degree of saturation have a great impact on the cytotoxic activity of flavoglaucin [59].

## 4. Materials and Methods

### 4.1. Chemicals

Ethyl acetate, cyclohexane, dichloromethane, methanol, and acetonitrile of HPLC grade (all with purity ≥ 99.8%) were purchased from Carlo Erba (Reagents (Val de Reuil, France)). Formic acid (≥99.3%) was purchased from VWR International (Fontenay-sous-Bois, France).

TLC sheet Reversed phase C18 (20–40 µm) was purchased from A.I.T France (Houilles, France).

TLC sheet ALUGRAM XTRA SIL G/UV254, 2 mm) and medium culture potato dextrose agar were purchased from Dominique Dutscher SAS (Brumath, France).

Sephadex LH-20 was purchased from Sigma Aldrich (Darmstadt, Germany). Silica gel was purchased from Merck (Darmstadt, Germany).

### 4.2. General Experimental Procedures

^1^H NMR and ^13^C NMR spectra were acquired with a Bruker Avance III HD spectrometer at 500 and 125 MHz, respectively, on the BISCEm Platform.

HPLC analyses were performed on an HPLC-UV system comprising a Shimadzu LC-20AB pump, an SIL-20AC autosampler, a CTO-10AS oven column, and for detection, a Shimadzu SPD-M20A photodiode array (Shimadzu Corp., Kyoto, Japan). The HPLC instrument was monitored by Shimadzu LCMS LabSolution software (Version: 5.127SP1).

The HPLC analytical column used was a reverse-phase Nucleodur C18 column (particle size of 10 µm; 250 mm × 4.6 mm I.D.). The mobile phase consisted of ultrapure H_2_O with 0.1% formic acid (A) and ACN with 0.1% formic acid (B). The gradient was delivered at a flow rate of 1 mL/min, and the injection volume was 10 μL. The column oven temperature was 40 °C. For UV–visible detection, the wavelength sweep was set between 200 and 400 nm.

The semi-preparative HPLC was a Waters 600 pump with a Waters 996 photodiode array detector. The column used was a reverse-phase Nucleodur C18 column (particle size of 10 µm; 250 mm × 21 mm I.D.). The mobile phase consisted of ultrapure H_2_O with 0.1% formic acid (A) and ACN with 0.1% formic acid (B). The gradient was delivered at a flow rate of 20 mL/min, the samples were prepared at a concentration of 10 mg/mL, and the injection volume was 250 μL at room temperature. For UV–visible detection, the wavelength was set to 254 nm. Empower software (version: 6.20.00.00) was used for spectra analysis.

MPLC was carried out by using Buchi pump model C-810 with silica gel 60 (40–63 µm; Merck).

### 4.3. LC/MS-MS Analyses

LC/MS-MS analyses were performed by using a Nexera X2 LC40 system (Shimadzu Corporation, Noisiel, France) coupled to a DAD covering the full range of acquisition (190–800 nm). It was interfaced with a quadrupole time-of-flight (QTOF) mass spectrometer (TripleTOF^®^ 5600+; Sciex, Concord, Vaughan, OR, Canada), equipped with a DuoSprayTM ion source. Separation was achieved on an ATLANTIS T3 100 Å (2.1 mm × 100 mm) with particle size of 5 µm. The column was thermostated at 40 °C, and a solvent gradient (200 µL/min) using water + 0.1% FA (solvent A) and acetonitrile + 0.1% FA (solvent B) was applied from 5% to 95% of solvent B in 20 min. The samples were reconstituted at 1 mg/mL with methanol, and 5 µL was injected. Mass experiments were recorded with an ESI ion source in positive and negative modes by using the following parameters: declustering potential (DP) of 50 V and −50 V, respectively; collision energy (CE) of 30 and −30 ± 15 V, respectively. Acquisition was performed in DDA (Data-Dependent Acquisition) mode, in which the TripleTOF 5600+ continuously switched between a survey 200 ms scan acquired in the TOF-MS mode (from 50 to 1500 *m*/*z*) and up to 15 dependent scans of 100 ms scan required in TOF-MS/MS mode (from 10 to 2000 *m*/*z*).

### 4.4. Data Treatment (Molecular Network Construction)

LC/MS-MS profiles were downloaded in MZmine2 software (version 2.53) in *.mzXML format. Peak detection in positive mode was performed by using the “mass detection1” (MS1) algorithm with a noise level of “1E3” in centroid mode. The same parameters were used for “mass detection2” (MS2).

Then, chromatograms were constructed for all detected ions with the “ADAP chromatogram builder” algorithm with a min group size scan of 5, a group intensity threshold of 500, a min highest intensity of 1000, and an *m*/*z* tolerance of 0.005 or 20 ppm. Peak deconvolution was applied to generate chromatograms with the “Baseline cut-off” algorithm with the following parameters: min peak height of 2000, peak duration range from 0 to 2, and baseline level 1E3; the *m*/*z* range for MS2 scan pairing (Da) of 0.02 was ticked, and the RT range of 0.5 was also ticked. Isotope removal was carried out with the isotopic peaks grouper by using the following parameters: *m*/*z* tolerance of 0.005 or 10 ppm, RT tolerance of 0.2 min, and maximum charge of 2; the most representative ion kept was the most intense. Alignment was then performed with an *m*/*z* tolerance of 0.005 to 10 ppm and an RT tolerance of 0.2 min, with an RT after correction of 0.1, an RANSAC iterative of 0, a min number of points of 50%, and a threshold value of 0.2; the required same charge state was ticked. An *.csv file was generated, gathering 1561 peaks with the corresponding MS2 data. The molecular network was built by using both MetGem and the GNPS platform. The following parameters were applied for MetGem: the cosine score was set to 0.7, MS1 data were ticked, *m*/*z* tolerance was set to 0.02 Da, the minimum matched peaks was set to 4, and the min intensity was 0.

### 4.5. Extraction Isolation and Purification

The lichen *Xanthoparmelia conspersa* was collected in May 2020 in the Vaseix forest in Limoges (45.832310, 1.177927). The isolation and identification of the *Aspergillus* sp. isolate were performed as previously described [60]. Briefly, the ITS sequence obtained after DNA extraction and purification, PCR amplification, and sequencing was compared to fungal sequences present in the NCBI GenBank database. A similarity of 97% allowed only for identification at the genus level.

This ELF was stored at −80 °C at the LABC*i*S laboratory under code XC04.

The XC04 endolichenic fungus was grown on solid potato dextrose agar (234 g in 6 L of distilled water, autoclaved for 20 min at 121 °C) in 230 square petri dishes for 21 days in a temperature-controlled chamber at 25 °C.

The fungal cultures were extracted with ethyl acetate (2.5 L) and homogenized for 48 h for 3 times. The combined extracts were then evaporated under pressure, resulting in a crude extract of 5 g. The latter was subjected to liquid–liquid partition between n-hexane (100 mL) and aqueous methanol (90% MeOH/10% H_2_O) (200 mL).

The intermediate subfraction obtained (203.3 mg) was fractionated by Sephadex LH-20 gel and eluted with CHCl_3_/MeOH 2:1, resulting in 8 fractions (F1−F8), which were then grouped according to their profile in thin-layer chromatography. Fractions 5–8 (11.6 mg) were purified by preparative TLC, eluted with CH_2_Cl_2_/MeOH 98:2, to give compound **1** (1 mg).

Fraction 4 (48 mg) was subjected to reverse-phase silica column separation by Flash C-pure by using a gradient of ACN: H_2_O from 65:35 to 85:15, yielding three fractions (4-A, 4-B, and 4-C).

Fraction 4-A was then separated by a semi-preparative HPLC C-18 column by using ACN:H_2_O from 70:30 to 100:0 at 20 mL/min for 21 min, which resulted in compound **2** (1.2 mg) and compound **3** (1 mg).

Fraction 4-B was separated by a semi-preparative HPLC C-18 column by using ACN:H_2_O from 70:30 to 100:0 at 20 mL/min for 21 min, which gave compound **4** (1.3 mg).

Fraction 4-C (10.8 mg) was separated by a semi-preparative HPLC C-18 column by using ACN:H_2_O from 70:30 to 100:0 at 20 mL/min for 21 min, yielding compound **4** (1.2 mg), compound **1** (3.8 mg), and compound **5** (2.1 mg).

The precipitate was purified by preparative TLC with CH_2_Cl_2_/MeOH (95/5) to give a mixture of compound **2** (4.7 mg) and compound **4** (1.7 mg), and pure compound **1** (10.9 mg).

### 4.6. Cell Culture and Treatments

The HT-29 human CRC cell line was from the American Type Culture Collection (ATCC-LGC Standards, Mosheim, France) used in LABC*i*S (Limoges, France). The culture medium used was Roswell Park Memorial Institute (RPMI, Gibco BRL–Fisher Scientific (Illkirch, France)) medium.

The human MDA-MB-231 (TNBC) and the human embryonic kidney non-cancerous HEK293 cell lines were also from ATCC and were used in the framework within the LIENSs laboratory (La Rochelle, France). The culture medium used was Dulbecco’s modified Eagle’s medium (DMEM) for both cell lines.

All culture media were supplemented with 10% fetal calf serum (FCS) and 1% penicillin–streptomycin antibiotic solution. RPMI medium was in addition supplemented with 1% glutamine.

Cell cultures were preserved in a humid atmosphere with 5% CO_2_ at 37 °C.

Cells were seeded in culture media and left to adhere for 24 h.

A stock solution of each extract and molecules was prepared at a concentration of 1 mg/mL in dimethyl sulfoxide (DMSO) and diluted in culture medium to obtain the de-sired test concentrations.

### 4.7. Cytotoxicity (MTT) Assay

Cell viability was measured by using the 3-(4,5-dimethylthiazol-2-yl)-2,5-diphenyltetrazolium bromide (MTT) assay (Sigma-Aldrich, Saint-Quentin-Fallavier, France). HT-29 cells were seeded in vitro in 96-well plates at 8000 cells per well. After 24 h of adhesion, the cells were treated with concentrations (0.5–40 µg/mL) of fungal extracts and molecules diluted in the culture medium. After 24 and 48 h of contact with the treatment, the absorbance was measured at 550 nm by a microplate reader (Multiskan FC, Thermo Scientific (Illkirch, France)). The results obtained were from 3 independent experiments.

MDA-MB-231 and HEK293 cells were seeded in vitro in 96-well plates at 10,000 cells per well and treated with different concentrations (0.5 µg/mL to 40 µg/mL) of fungal extracts.

### 4.8. DNA Fragmentation

The phenomenon of apoptosis was studied by quantifying the mono- and oligonucleosomes in the cytoplasmic fraction of HT-29 cell lysates after treatment by using the cell death ELISA (Cell Death Detection ELISA^PLUS^; Roche Diagnostics, Basel, Switzerland).

A total of 2 × 10^5^ cells of each condition were then obtained, and DNA fragmentation was measured as detailed below.

HT-29 cells were grown in T75 flasks. After treatment or not with IC_50_, IC_50_ × 2, and IC_50_ × 3 of the EtOAc extract (XC04 GP) and Echinulin, pellets of 2 × 10^5^ cells were obtained from the two pooled cell fractions (floating cells and adherent cells) after trypsin harvesting. The assay was performed on 20 µL of cell lysate of each condition placed into a streptavidin-coated microplate. A mixture (80 µL) of mouse monoclonal antibodies directed against DNA and histones diluted in incubation buffer was added to each well and then incubated under gentle shaking (300 rpm) for 2 h at room temperature. After incubation, the solution was removed, and the wells were rinsed three times with incubation buffer before adding 100 µL per well of substrate ABTS solution; afterwards, the microplate was incubated at 250 rpm for approximately 10–20 min.

The reaction was developed with the peroxidase substrate and stopped with ABTS stop solution (100 µL). The absorbance was measured at 405 nm and 490 nm (reference wavelength) with a microplate reader. The results were reported as n-fold compared with the control.

### 4.9. Statistical Analysis

One-way or two-way analysis of variance (ANOVA) was performed to identify significant differences between the control and experimental groups. All experimental data were acquired from at least three independent experiments. All statistical analyses were performed with GraphPad Prism 9.0.0 for Windows. A probability (*p*) value of <0.05 was considered statistically significant.

## 5. Conclusions

The chemical and biological potential of endolichenic fungi is confirmed through this study. A small number of annotated nodes was found in the metabolomic network analysis conducted on four extracts issued from an endolichenic fungus. However, the molecular network identified two intriguing and often biosynthesized compounds by the *Aspergillus* genus (flavoglaucin and echinulin derivatives). Considering the fact that no cytotoxicity was reported against healthy cells after treatment with *Aspergillus* sp. EtOAc extract, further studies on the activity of this extract against human chemoresistant cancers, such as TNBC and CRC, reveal a preference for colorectal cancer. Therefore, these cell lines were used to examine the isolated compounds. Their activity ranged from 1.7 µM to 44.84 µM, which is noteworthy. Additional biological tests of these molecules are needed to determine their signaling pathway.

## Figures and Tables

**Table 1:** Cell viability (MTT test) of the intermediate subfraction and precipitate on HT-29 cells at 24 and 48 h.

Subfractions	IC_50_ (µg/mL) at 24 h	IC_50_ (µg/mL) at 48 h
Hexane subfraction	39.5 ± 6.4	19.8 ± 1.9
Hydro-methanol subfraction	27.9 ± 3.6	36.9 ± 3.4
Intermediate subfraction	4.8 ± 1.1	1.8 ± 0.3
Precipitate	0.9 ± 0.6	0.8 ± 0.9

IC_50_: medium inhibitory concentration. Concentration that causes 50% of growth inhibition.

**Table 2:** Annotated compounds in the molecular network of extracts of *Aspergillus* sp.

*m*/*z* [M + H]^+^ Parent	Molecular Formula [M]	R*_t_* (min)	Annotation	Cosine Score	Database
180.0948	C_10_H_13_NO_2_	7.14	N-Acetyltyramine	0.84	Massbank/GNPS
185.1262	C_9_H_18_N_2_O_3_	7.30	Cyclo (L-ala-L-Leu)	0.83	GNPS
197.1269	C_10_H_16_N_2_O_2_	6.79	Cyclo (L-Val-L-Pro)	0.95	GNPS
211.1428	C_11_H_18_N_2_O_2_	7.56	Cyclo (L-Leu-L-Pro)	0.87	NIH
245.1347	C_14_H_16_N_2_O_2_	8.49	Cyclo (L-Phe-D-Pro)	0.96	GNPS
257.2447	C_16_H_32_O_2_	21.11	Pentadecanoic acid	0.83	GNPS
261.1587	C_15_H_20_N_2_O_2_	10.56	Cyclo (Phe-Leu)	0.89	GNPS
261.1243	C_14_H_16_N_2_O_3_	6.62	Cyclo (L-Tyr-L-Pro)	0.87	NIST
267.1638	C_17_H_24_O_4_	18.34	Tributylphosphate	0.72	GNPS
279.1617	C_20_H_30_O_4_	19.69	Dibutylphtalate	0.82	GNPS
281.2458	C_16_H_17_N_3_	22.63	Linoleic acid	0.83	Massbank
284.2899	C_18_H_37_NO	22.42	Octadecanamide	0.84	GNPS
305.2082	C_16_H_22_O_9_	21.70	Flavoglaucin	0.84	NIH
324.1679	C_19_H_21_N_3_O_2_	12.44	Neoechinulin A	0.84	GNPS
326.1908	C_19_H_23_N_3_O_2_	11.60	Preechinulin	0.82	NIH
331.2803	C_26_H_36_O_6_	22.80	1-Hexadecanoyl-sn-glycerol	0.86	NIH/GNPS
348.3072	C_29_H_46_O_11_, [M + NH_4_]^+^	22.86	2,3-Dihydroxypropyl hexadecanoate	0.79	NIH
359.3043	C_28_H_37_NO_9_	24.86	Glycerol 1-stearate	0.78	GNPS
462.3056	C_29_H_39_N_3_O_2_	21.85	Echinulin	0.80	GNPS
563.5507	C_18_H_37_NO, [2M + H]^+^	18.89	9-Octadecenamide	0.80	GNPS
567.5745	C_18_H_37_NO, [2M + H]^+^	22.42	Octadecanamide	0.80	GNPS
675.6664	C_22_H_41_NO_3,_ [2M + H]^+^	25.94	13-Docosenamide	0.81	GNPS

**Table 3:** Growth inhibition of isolated compounds from *Aspergillus* sp. on HT-29 cells.

Molecules	IC_50_ (µM) at 24 h	IC_50_ (µM) at 48 h
Echinulin (**1**)	1.51 ± 0.1	1.73 ± 0.1
8-Hydroxyechinulin (**2**)	7.12 ± 0.7	8.80 ± 1.1
Didehydroechinulin B (**3**)	>50	44.84 ± 0.7
Tardioxopiperazine B (**4**)	11.90 ± 0.8	13.70 ± 0.0
Flavoglaucin (**5**)	9.17 ± 1.4	34.40 ± 0.9
5-Fluorouracil	>50	17.30 ± 1.4
Irinotecan	44.50 ± 1.2	28.00 ± 0.4

## Data Availability

The original contributions presented in the study are included in the article, further inquiries can be directed to the corresponding author/s.

## References

[1] Ferlay J., Colombet M., Soerjomataram I., Parkin D.M., Piñeros M., Znaor A., Bray F. (2021). Cancer Statistics for the Year 2020: An Overview. Int. J. Cancer.

[2] Matthews H.K., Bertoli C., de Bruin R.A.M. (2022). Cell Cycle Control in Cancer. Nat. Rev. Mol. Cell Biol..

[3] Sung H., Ferlay J., Siegel R.L., Laversanne M., Soerjomataram I., Jemal A., Bray F. (2021). Global Cancer Statistics 2020: GLOBOCAN Estimates of Incidence and Mortality Worldwide for 36 Cancers in 185 Countries. CA. Cancer J. Clin..

[4] Catalano A., Iacopetta D., Ceramella J., Mariconda A., Rosano C., Scumaci D., Saturnino C., Longo P., Sinicropi M.S. (2022). New Achievements for the Treatment of Triple-Negative Breast Cancer. Appl. Sci..

[5] Simelane N.W.N., Ann Kruger C., Abrahamse H. (2020). Photodynamic Diagnosis and Photodynamic Therapy of Colorectal Cancer In Vitro and In Vivo. RSC Adv..

[6] Khalifa S.A.M., Elias N., Farag M.A., Chen L., Saeed A., Hegazy M.-E.F., Moustafa M.S., Abd El-Wahed A., Al-Mousawi S.M., Musharraf S.G. (2019). Marine Natural Products: A Source of Novel Anticancer Drugs. Mar. Drugs.

[7] Liu S., Khan A.R., Yang X., Dong B., Ji J., Zhai G. (2021). The Reversal of Chemotherapy-Induced Multidrug Resistance by Nanomedicine for Cancer Therapy. J. Controlled Release.

[8] Naeem A., Hu P., Yang M., Zhang J., Liu Y., Zhu W., Zheng Q. (2022). Natural Products as Anticancer Agents: Current Status and Future Perspectives. Molecules.

[9] Newman D.J., Cragg G.M. (2012). Natural Products As Sources of New Drugs over the 30 Years from 1981 to 2010. J. Nat. Prod..

[10] Newman D.J., Cragg G.M. (2016). Natural Products as Sources of New Drugs from 1981 to 2014. J. Nat. Prod..

[11] White J. (2009). Drug Addiction: From Basic Research to Therapy. Drug Alcohol Rev..

[12] Nobili S., Lippi D., Witort E., Donnini M., Bausi L., Mini E., Capaccioli S. (2009). Natural Compounds for Cancer Treatment and Prevention. Pharmacol. Res..

[13] Dong J., Qian Y., Zhang G., Lu L., Zhang S., Ji G., Zhao A., Xu H. (2022). Can Natural Products Be Used to Overcome the Limitations of Colorectal Cancer Immunotherapy?. Front. Oncol..

[14] Zhang W., Ran Q., Li H., Lou H. (2024). Endolichenic Fungi: A Promising Medicinal Microbial Resource to Discover Bioactive Natural Molecules—An Update. J. Fungi.

[15] Podojil M., Sedmera P., Vokoun J., Betina V., Baráthová H., Ďuračková Z., Horáková K., Nemec P. (1978). *Eurotium (Aspergillus) repens* Metabolites and Their Biological Activity. Folia Microbiol..

[16] Hawksworth D.L., Grube M. (2020). Lichens Redefined as Complex Ecosystems. New Phytol..

[17] U’Ren J.M., Lutzoni F., Miadlikowska J., Arnold A.E. (2010). Community Analysis Reveals Close Affinities between Endophytic and Endolichenic Fungi in Mosses and Lichens. Microb. Ecol..

[18] Kellogg J.J., Raja H.A. (2017). Endolichenic Fungi: A New Source of Rich Bioactive Secondary Metabolites on the Horizon. Phytochem. Rev..

[19] Paranagama P.A., Wijeratne E.K., Burns A.M., Marron M.T., Gunatilaka M.K., Arnold A.E., Gunatilaka A.L. (2007). Heptaketides from *Corynespora* sp. Inhabiting the Cavern Beard Lichen, *Usnea cavernosa*: First Report of Metabolites of an Endolichenic Fungus. J. Nat. Prod..

[20] Ding G., Li Y., Fu S., Liu S., Wei J., Che Y. (2009). Ambuic Acid and Torreyanic Acid Derivatives from the Endolichenic Fungus *Pestalotiopsis* sp.. J. Nat. Prod..

[21] Zhang F., Liu S., Lu X., Guo L., Zhang H., Che Y. (2009). Allenyl and Alkynyl Phenyl Ethers from the Endolichenic Fungus *Neurospora terricola*. J. Nat. Prod..

[22] Li X.-B., Li L., Zhu R.-X., Li W., Chang W.-Q., Zhang L.-L., Wang X.-N., Zhao Z.-T., Lou H.-X. (2015). Tetramic Acids and Pyridone Alkaloids from the Endolichenic Fungus *Tolypocladium cylindrosporum*. J. Nat. Prod..

[23] Graham F.L., Smiley J., Russell W.C., Nairn R. (1977). Characteristics of a Human Cell Line Transformed by DNA from Human Adenovirus Type 5. J. Gen. Virol..

[24] Li S., Liu X., Gu Q., Yu X. (2024). Isolation and Identification of Indole Alkaloids from *Aspergillus amstelodami* BSX001 and Optimization of Ultrasound-Assisted Extraction of Neoechinulin A. Microorganisms.

[25] Almeida A.P., Dethoup T., Singburaudom N., Lima R., Vasconcelos M.H., Pinto M., Kijjoa A. (2010). The in Vitro Anticancer Activity of the Crude Extract of the Sponge-Associated Fungus *Eurotium cristatum* and Its Secondary Metabolites. J. Nat. Pharm..

[26] Lv D., Xia J., Guan X., Lai Q., Zhang B., Lin J., Shao Z., Luo S., Zhangsun D., Qin J.-J. (2022). Indole Diketopiperazine Alkaloids Isolated From the Marine-Derived Fungus *Aspergillus chevalieri* MCCC M23426. Front. Microbiol..

[27] Fujimoto H., Fujimaki T., Okuyama E., Yamazaki M. (1999). Immunomodulatory Constituents from an Ascomycete, *Microascus tardifaciens*. Chem. Pharm. Bull..

[28] Gao H., Wang Y., Luo Q., Yang L., He X., Wu J., Kachanuban K., Wilaipun P., Zhu W., Wang Y. (2021). Bioactive Metabolites from Acid-Tolerant Fungi in a Thai Mangrove Sediment. Front. Microbiol..

[29] Ségal-Bendirdjian E. (1999). Mort Cellulaire: Signalisation et Exécution de l’apoptose. Hématologie.

[30] Huang M., Lu J.-J., Ding J. (2021). Natural Products in Cancer Therapy: Past, Present and Future. Nat. Prod. Bioprospecting.

[31] Yang Y., Bae W.K., Nam S.-J., Jeong M.-H., Zhou R., Park S.-Y., Taş İ., Hwang Y.-H., Park M.-S., Chung I.J. (2018). Acetonic Extracts of the Endolichenic Fungus EL002332 Isolated from *Endocarpon pusillum* Exhibits Anticancer Activity in Human Gastric Cancer Cells. Phytomedicine.

[32] Nguyen T.T., Yoon S., Yang Y., Lee H.-B., Oh S., Jeong M.-H., Kim J.-J., Yee S.-T., Crişan F., Moon C. (2014). Lichen Secondary Metabolites in *Flavocetraria cucullata* Exhibit Anti-Cancer Effects on Human Cancer Cells through the Induction of Apoptosis and Suppression of Tumorigenic Potentials. PLoS ONE.

[33] Yang Y., Park S.-Y., Nguyen T.T., Yu Y.H., Nguyen T.V., Sun E.G., Udeni J., Jeong M.-H., Pereira I., Moon C. (2015). Lichen Secondary Metabolite, Physciosporin, Inhibits Lung Cancer Cell Motility. PLoS ONE.

[34] Taş İ., Han J., Park S.-Y., Yang Y., Zhou R., Gamage C.D.B., Van Nguyen T., Lee J.-Y., Choi Y.J., Yu Y.H. (2019). Physciosporin Suppresses the Proliferation, Motility and Tumourigenesis of Colorectal Cancer Cells. Phytomedicine.

[35] Taş İ., Varlı M., Son Y., Han J., Kwak D., Yang Y., Zhou R., Gamage C.D.B., Pulat S., Park S.-Y. (2021). Physciosporin Suppresses Mitochondrial Respiration, Aerobic Glycolysis, and Tumorigenesis in Breast Cancer. Phytomedicine.

[36] Yang Y., Bhosle S.R., Yu Y.H., Park S.-Y., Zhou R., Taş İ., Gamage C.D.B., Kim K.K., Pereira I., Hur J.-S. (2018). Tumidulin, a Lichen Secondary Metabolite, Decreases the Stemness Potential of Colorectal Cancer Cells. Molecules.

[37] Zhou R., Yang Y., Park S.-Y., Nguyen T.T., Seo Y.-W., Lee K.H., Lee J.H., Kim K.K., Hur J.-S., Kim H. (2017). The Lichen Secondary Metabolite Atranorin Suppresses Lung Cancer Cell Motility and Tumorigenesis. Sci. Rep..

[38] Frisvad J.C., Larsen T.O. (2015). Chemodiversity in the Genus *Aspergillus*. Appl. Microbiol. Biotechnol..

[39] Wong R.S. (2011). Apoptosis in Cancer: From Pathogenesis to Treatment. J. Exp. Clin. Cancer Res..

[40] Elmore S. (2007). Apoptosis: A Review of Programmed Cell Death. Toxicol. Pathol..

[41] Zhu J., Song L., Shen S., Fu W., Zhu Y., Liu L. (2023). Bioactive Alkaloids as Secondary Metabolites from Plant Endophytic *Aspergillus* Genus. Molecules.

[42] Quilico A., Panizzi L., Mugnaini E. (1949). Structure of Flavoglaucin and Auroglaucin. Nature.

[43] Gao J., León F., Radwan M.M., Dale O.R., Husni A.S., Manly S.P., Lupien S., Wang X., Hill R.A., Dugan F.M. (2011). Benzyl Derivatives with In Vitro Binding Affinity for Human Opioid and Cannabinoid Receptors from the Fungus *Eurotium repens*. J. Nat. Prod..

[44] Ali M., Mohammed N., Alnaqeeb M.A., Hassan R.A., Ahmad H.S. (1989). Toxicity of Echinulin from *Aspergillus chevalieri* in Rabbits. Toxicol. Lett..

[45] Ghafouri-Fard S., Abak A., Tondro Anamag F., Shoorei H., Fattahi F., Javadinia S.A., Basiri A., Taheri M. (2021). 5-Fluorouracil: A Narrative Review on the Role of Regulatory Mechanisms in Driving Resistance to This Chemotherapeutic Agent. Front. Oncol..

[46] Longley D.B., Harkin D.P., Johnston P.G. (2003). 5-Fluorouracil: Mechanisms of Action and Clinical Strategies. Nat. Rev. Cancer.

[47] Bailly C. (2019). Irinotecan: 25 Years of Cancer Treatment. Pharmacol. Res..

[48] Yan Z.-F., Lin P., Kook M., Yi T.-H., Li C.-T. (2017). Immune Activation Effects of *Eurotium cristatum* on T Cells through NF-ΚB Signaling Pathways in Humans. Food Agric. Immunol..

[49] Smetanina O.F., Yurchenko A.N., Girich (Ivanets) E.V., Trinh P.T.H., Antonov A.S., Dyshlovoy S.A., von Amsberg G., Kim N.Y., Chingizova E.A., Pislyagin E.A. (2020). Biologically Active Echinulin-Related Indolediketopiperazines from the Marine Sediment-Derived Fungus *Aspergillus niveoglaucus*. Molecules.

[50] Zhong W.-M., Wang J.-F., Shi X.-F., Wei X.-Y., Chen Y.-C., Zeng Q., Xiang Y., Chen X.-Y., Tian X.-P., Xiao Z.-H. (2018). Eurotiumins A–E, Five New Alkaloids from the Marine-Derived Fungus *Eurotium* sp. SCSIO F452. Mar. Drugs.

[51] Zhou L., Zhu T., Cai S., Gu Q., Li D. (2010). Three New Indole-Containing Diketopiperazine Alkaloids from a Deep-Ocean Sediment Derived Fungus *Penicillium griseofulvum*. Helv. Chim. Acta.

[52] Gao H., Liu W., Zhu T., Mo X., Mándi A., Kurtán T., Li J., Ai J., Gu Q., Li D. (2012). Diketopiperazine Alkaloids from a Mangrove Rhizosphere Soil Derived Fungus *Aspergillus effuses* H1-1. Org. Biomol. Chem..

[53] Gao H., Zhu T., Li D., Gu Q., Liu W. (2013). Prenylated Indole Diketopiperazine Alkaloids from a Mangrove Rhizosphere Soil Derived Fungus *Aspergillus effuses* H1-1. Arch. Pharm. Res..

[54] Elsbaey M., Sallam A., El-Metwally M., Nagata M., Tanaka C., Shimizu K., Miyamoto T. (2019). Melanogenesis Inhibitors from the Endophytic Fungus *Aspergillus amstelodami*. Chem. Biodivers..

[55] Kamauchi H., Kinoshita K., Sugita T., Koyama K. (2016). Conditional Changes Enhanced Production of Bioactive Metabolites of Marine Derived Fungus *Eurotium rubrum*. Bioorg. Med. Chem. Lett..

[56] Fathallah N., Raafat M.M., Issa M.Y., Abdel-Aziz M.M., Bishr M., Abdelkawy M.A., Salama O. (2019). Bio-Guided Fractionation of Prenylated Benzaldehyde Derivatives as Potent Antimicrobial and Antibiofilm from *Ammi majus* L. Fruits-Associated *Aspergillus amstelodami*. Molecules.

[57] Luo Y., Luo X., Zhang T., Li S., Liu S., Ma Y., Wang Z., Jin X., Liu J., Wang X. (2022). Anti-Tumor Secondary Metabolites Originating from Fungi in the South China Sea’s Mangrove Ecosystem. Bioengineering.

[58] Yoshimi N., Wang A., Morishita Y., Tanaka T., Sugie S., Kawai K., Yamahara J., Mori H. (1992). Modifying Effects of Fungal and Herb Metabolites on Azoxymethane-induced Intestinal Carcinogenesis in Rats. Jpn. J. Cancer Res..

[59] Lin L.-B., Gao Y.-Q., Han R., Xiao J., Wang Y.-M., Zhang Q., Zhai Y.-J., Han W.-B., Li W.-L., Gao J.-M. (2021). Alkylated Salicylaldehydes and Prenylated Indole Alkaloids from the Endolichenic Fungus *Aspergillus chevalieri* and Their Bioactivities. J. Agric. Food Chem..

[60] Lagarde A., Jargeat P., Roy M., Girardot M., Imbert C., Millot M., Mambu L. (2018). Fungal Communities Associated with *Evernia prunastr*i, *Ramalina fastigiata* and *Pleurosticta acetabulum*: Three Epiphytic Lichens Potentially Active against *Candida* Biofilms. Microbiol. Res..

